# Antimicrobial resistance and molecular detection of extended spectrum β-lactamase producing *Escherichia coli* isolates from raw meat in Greater Accra region, Ghana

**DOI:** 10.1186/s12866-020-01935-z

**Published:** 2020-08-14

**Authors:** Esther Dsani, Edwin Andrews Afari, Anthony Danso-Appiah, Ernest Kenu, Basil Benduri Kaburi, Beverly Egyir

**Affiliations:** 1 Department of Epidemiology and Disease Control, School of Public Health, University of Ghana, Accra, Ghana; 2 Veterinary Services Directorate of the Ministry of Food and Agriculture, Accra, Ghana; 3 Bacteriology Department, Noguchi Memorial Institute for Medical Research, University of Ghana, Accra, Ghana

**Keywords:** Antibiotic resistance, *E. coli*, Raw meat, ESBL, Ghana

## Abstract

**Background:**

Typically, raw meat can be contaminated with antibiotic resistant pathogens at unhygienic slaughter and sale points. Consumption of meat contaminated with antibiotic resistant *E. coli* is associated with grave health care consequences. The aim of this study was to determine the microbial quality of raw meat, the antimicrobial susceptibility and Extended Spectrum Beta Lactamase (ESBL) production in *E. coli* isolates from raw meat.

**Results:**

Total Plate Counts exceeded the acceptable limit of 5.0 log CFU/ cm^2^ in 60.5% (124/205) of raw meat samples. Total Coliform Counts in 70.7% (145/205) of samples were in excess of the acceptable limit of 2.5 log CFU/cm^2^. *E. coli* was detected in about half of raw meat samples (48%), ranging from 9.5–79.0% among the slaughter sites. Isolates were susceptible to meropenem (100%), ceftriaxone (99%), cefotaxime (98%), chloramphenicol (97%), gentamycin (97%), ciprofloxacin (92%) and amikacin (92%), but resistant to ampicillin (57%), tetracycline (45%), sulfamethoxazole-trimethoprim (21%) and cefuroxime (17%). Multi-drug resistance (MDR) was identified in 22% of the isolates. The *bla*_*TEM gene*_ was detected in 4% (4/98) of *E. coli* isolates in this study.

**Conclusion:**

The levels of microbial contamination of raw meat in this study were unacceptable. Meat handlers and consumers are at risk of foodborne infections from *E. coli* including ESBL producing *E. coli* that are resistant to most antibiotics in use. We recommend an enhanced surveillance for antibiotic resistance in food products for the early detection of emerging resistant bacteria species in the food chain.

## Background

The emergence of antibiotic resistance in bacteria commonly found in food animals has garnered attention globally for its potential contribution to human colonization with antibiotic resistant bacteria. Antibiotics are used in food animals for therapeutic and non-therapeutic purposes. The continuous use of antibiotics in food animal production is cited as a major determinant for carriage of antibiotic resistant bacteria in food animals [1]. A proportion of antibiotic classes are used in both animals and humans, creating the need to monitor the spread of resistant bacteria from animals to humans at all stages of the transmission pathway [2].

Food products of animal origin contaminated with drug resistant bacteria may provide a direct route for human colonization [3]. Meat is an important part of the Ghanaian diet and is often sourced from open markets and cold stores. Chicken, beef and chevon are the most patronized meat types while pork and mutton have low patronage [4]. Although selective pressure due to antibiotic use in primary production is considered as a major source of antibiotic resistant bacteria in livestock products, sanitary conditions at slaughter, sale and processing points may affect the pattern and intensity of spread along the food chain [5, 6]. *Escherichia coli* is a common colonizer of the intestinal tract of humans and animals, and is widely known to cause bacteremia in humans [7]. The presence of pathogenic strains of *E. coli* in meat and dairy products has been associated with foodborne disease outbreaks in humans [8].

Extended spectrum beta lactamase (ESBL), an enzyme produced by some gram negative bacteria, poses a peculiar challenge for treatment as it confers resistance to penicillins, cephalosporins and monobactams [9]. Extended spectrum TEM-, SHV- and CTX-M type enzymes have been detected in *Enterobacteriaceae* from retail food [10]. ESBL-producing *E. coli* have been reported in patients with urinary tract infections worldwide [11]. The contribution of the food chain to the occurrence of ESBL bacteria in humans is widely debated with fecal-oral and nosocomial transmissions largely influencing its emergence and spread in humans [12]. Monitoring the trend of spread of ESBL positive *E. coli* through the food chain is necessary in settings where humans have routine contact with livestock and their products, because of the increased risk of spread of resistance genes in these interactions.

In Ghana, it has been found that, resistance in *Enterobacteriaceae* to commonly used antibiotic agents is widespread [13]. Research evidence on microbial contamination of meat and their antibiotic resistance patterns is limited and thus, it is difficult to assess the risk posed to human health through the food chain. Yet, such evidence is critical for guiding future antimicrobial therapy. The aim of this study was to determine the level of microbial contamination of raw meat, presence of *E. coli* and their resistance to commonly prescribed antibiotics for human infections. This study also detected the occurrence of ESBL production among *E. coli* isolates recovered from raw meat.

## Results

### Microbial contamination of raw meat

**Fig. 1:**
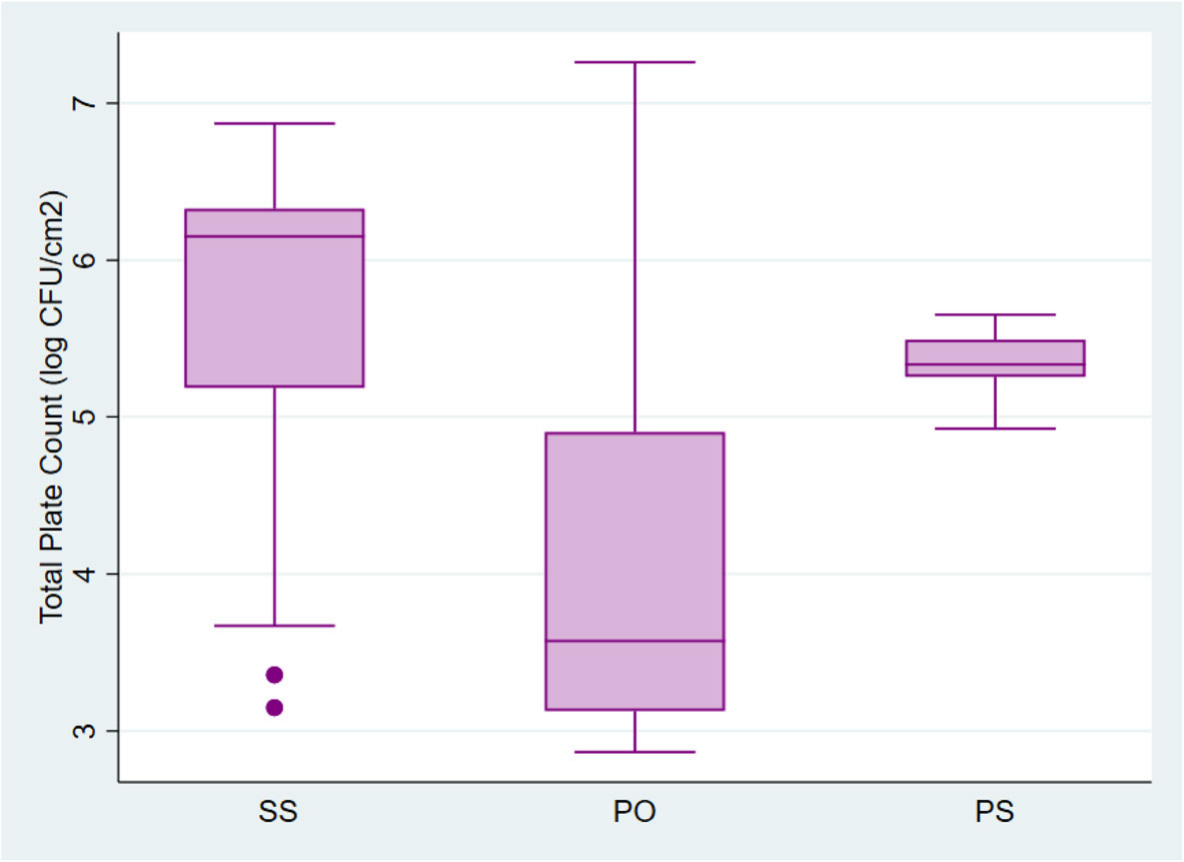
Distribution of Total Plate Counts among the three slaughter sites. SS- Slaughter slab, PO- Privately-owned slaughterhouse, PS- Public slaughterhouse

**Fig. 2:**
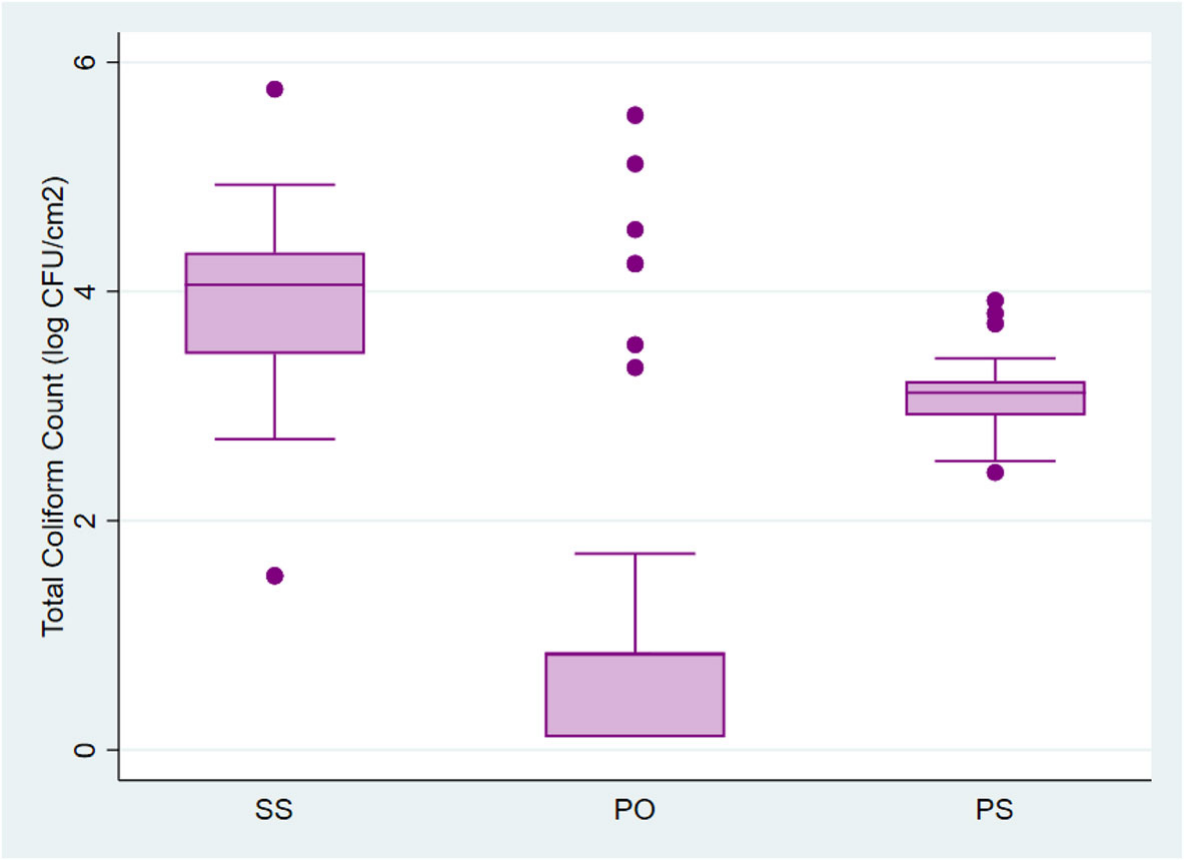
Distribution of Total Coliform Counts among the three slaughter sites. SS- Slaughter slab, PO- Privately-owned slaughterhouse, PS- Public slaughterhouse

Overall, 124 (60.5%) samples from raw meat in this study exceeded the maximum limit of 5.0 log CFU/cm^2^, for total aerobic counts for cattle, sheep and goat carcasses set by the Ghana Standards Authority and the European Commission Regulation (EC) on the microbial criteria for food stuffs. Total Plate Counts for all 205 samples ranged from 2.86 log CFU/cm^2^ to 7.26 log CFU/cm^2^ with a median of 5.28 log CFU/cm^2^ (Table 1). More than half of all samples (70.7%) had coliform counts exceeding the acceptable limit of 2.5 log CFU/cm^2^. Total coliform counts ranged from 0.11 log CFU/cm^2^ to 5.76 log CFU/cm^2^ with a median of 3.12 log CFU/cm^2^ (Table 1). The Plate Count and Coliform Count distribution among the three slaughter sites is shown in Figs. 1 and 2. The Kruskal-Wallis test showed that there was a statistically significant difference in total plate counts between the three slaughter sites, χ^2^ (2) =73.81, *p* = 0.0001. The distribution of coliforms in samples from the three sites were also found to differ significantly, χ^2^ (2) =110.94, p = 0.0001.

**Table 1:** Microbiological quality of raw meat samples from selected slaughterhouses in the Greater Accra region of Ghana

Slaughterhouse	Median count in log CFU/cm^**2**^ (range)	
	Total Plate Count	Total Coliform Count
**PS**	5.33 (4.92–5.65)	3.12 (2.42–3.92)
**SS**	6.15 (3.15–6.87)	4.05 (1.52–5.76)
**PO**	3.57 (2.86–7.26)	0.84 (0.11–5.54)
**Total (all sites)**	5.28 (2.86–7.26)	3.12 (0.11–5.76)

*PS* Public slaughterhouse, *SS* Slaughter slab, *PO* Privately-owned slaughterhouse

### Contamination of raw meat with *E. coli*

*E. coli* was detected in 48% (98/205) of all samples. The proportion of meat contaminated with *E. coli* was 79% (50/63) at the public slaughterhouse, 53% (42/79) at the slaughter slab and 9.5% (6/63) at the privately-owned slaughterhouse (Table 2). The rate of *E. coli* contamination was 61.7% (50/81) in samples obtained from beef, 40.7% (44/108) for chevon and 25% (4/16) for mutton. The results of the chi-square test showed that there was a significant difference in the proportions of *E. coli* contaminated meat among the slaughter sites (*p* < 0.05).

**Table 2:** Distribution of *E. coli* isolated from meat at slaughterhouses

Meat type	PS		SS		PO		Total	
	Samples tested	*E. coli* positive	Samples tested	*E. coli* positive	Samples tested	*E. coli* positive	Samples tested	*E. coli* positive
Beef	54	42 (78%)	24	8 (33%)	3	–	81	50 (61.7%)
Mutton	–	–	9	3 (33%)	7	1 (14%)	16	4 (25.0%)
Chevon	9	8 (89%)	46	31 (67%)	53	5 (9%)	108	44 (40.7%)
Total	63	50 (79%)	79	42 (53%)	63	6 (9.5%)	205	98 (48.0%)

*PS* Public slaughterhouse, *SS* Slaughter slab, *PO* Privately-owned slaughterhouse

### Antimicrobial susceptibility and detection of ESBL production

**Fig. 3:**
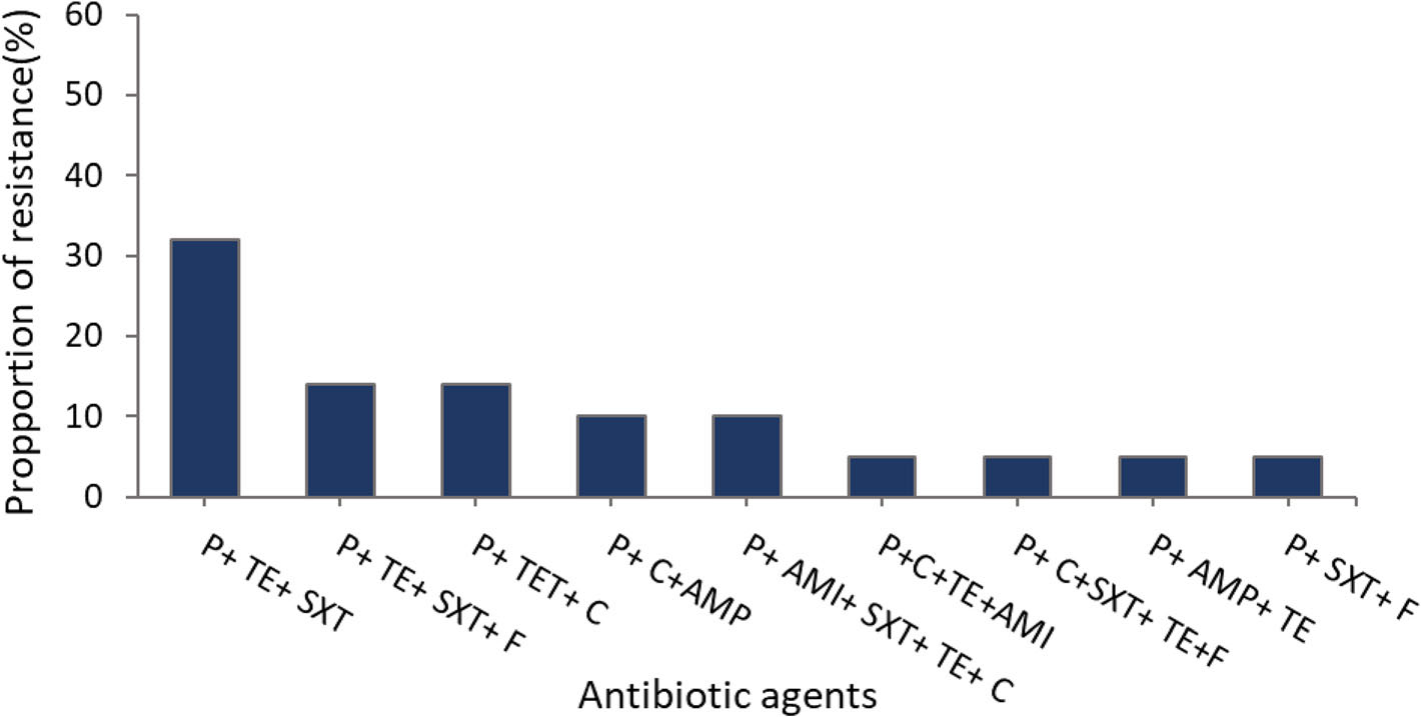
Multidrug resistance profiles of *E. coli* isolates. P-Penicillin, TE- Tetracycline, SXT-Sulphamethoxazole/ Trimethoprim, AMI- Aminoglycoside, C-Cephalosporin, F-Quinolones/Fluoroquinolones, AMP-Amphenicol

*E. coli* isolates recovered were frequently resistant to ampicillin (57%), tetracycline (45%), cefuroxime (21%), and sulphamethoxazole/trimethoprim (SXT) (17%) (Table 3). High rates of susceptibility were observed for ceftriaxone (99%), cefotaxime (98%), chloramphenicol (97%), gentamycin (97%), ciprofloxacin (92%) and amikacin (92%) (Table 3). All isolates were susceptible to meropenem. Of the 98 *E. coli* isolates tested, 84% (82/98) showed resistance to at least one out of the eleven antimicrobial agents. In all, 22% (22/98) of the isolates were multidrug resistant. Among the multidrug resistant *E. coli*, concurrent resistance to penicillin, tetracycline and SXT (32%), was most common (Fig. 3). Other patterns observed were resistance to penicillin, tetracycline, SXT, and fluoroquinolone class of antibiotics (14%), penicillin, tetracycline and cephalosporin class of antibiotics (14%), penicillin, tetracycline and amphenicols (10%) and penicillin, aminoglycoside, SXT, tetracycline and cephalosporin (10%). Most of the MDR patterns observed in the *E. coli* isolates (95%) showed resistance to penicillin and tetracycline class of antibiotics in addition to other antimicrobial classes. Out of the 98 *E. coli* isolates recovered from raw meat, 14 isolates were presumed to be ESBL producers based on the results of the combination disk test. Out of these, four [4] *E. coli* isolates were found to have the *bla*_*TEM*_ *gene.* Majority of *E. coli* isolates found to have the *bla*_*TEM*_ *gene* (3/4), showed phenotypic resistance to ampicillin, amikacin, tetracycline, SXT and cefuroxime. *Bla*_*SHV*,_ and *bla*_*CTX-M*_ genes were not detected in the isolates screened.

**Table 3:** Antimicrobial resistance profile of *E. coli* isolates from raw meat at the selected slaughter sites in the Greater Accra region of Ghana

Antimicrobial agent	PS *n* = 50 (%)	SS *n* = 42, (%)	PO *n* = 6 (%)	Total *N* = 98, (%)
Ampicillin	23 (46)	33 (79)	0 (0)	56 (57)
Tetracycline	26 (52)	18 (43)	0 (0)	44 (45)
Cefuroxime	8 (16)	13 (30)	0 (0)	21 (21)
Sulphamethoxazole/Trimethoprim	10 (20)	7 (16)	0 (0)	17 (17)
Amikacin	2 (4)	6 (14)	0 (0)	8 (8)
Ciprofloxacin	4 (8)	3 (7)	1 (16)	8 (8)
Cefotaxime	1 (2)	1 (2)	0 (0)	2 (2.1)
Gentamycin	2 (4)	1 (2)	0 (0)	3 (3.1)
Chloramphenicol	2 (4)	1 (2)	0 (0)	3 (3.1)
Ceftriaxone	0 (0)	1 (2)	0 (0)	1 (1)
Meropenem	0 (0)	0 (0)	0 (0)	0 (0)

*n* Number of *E. coli* isolates, *PS* Public slaughterhouse, *SS* Slaughter slab, *PO* Privately-owned slaughterhouse

## Discussion

The study findings show that, in most cases (> 60%), raw meat that are ready for sale at the selected study sites had microbial loads exceeding the acceptable limit. The majority of slaughtering in Ghana is conducted at slaughterhouses and slabs with sub-standard conditions of slaughter and minimal oversight from food safety authorities [14]. The absence of hygiene standards at points of slaughter has been linked to increased microbial contamination of carcasses [15]. High coliform and plate counts observed at the public slaughterhouse and slaughter slab may stem from the introduction of contaminants from floor surfaces, hides and faeces of livestock in the absence of standard operating procedures that ensure sanitary standards are met in the slaughter environment. Contamination of meat in such situations may also be exacerbated by inadequate slaughter infrastructure that ensures that work areas are properly segregated [16]. The consequence for human health lies in the potential exposure of consumers to disease causing organisms and carriage of drug resistant bacteria [17].

*E. coli* is commonly used as an indicator organism for assessing food and water hygiene [18]. Although *E. coli* was detected in close to half of raw meat sampled, wide-ranging proportions of *E. coli* contamination was observed at the slaughter sites (9.5–79%). Previous studies on *E. coli* in beef from retail joints in Northern Ghana similarly reported wide- ranging values of 0–100% [19]. While high *E. coli* contamination rates often indicate poor slaughter hygiene, the presence of other environmental contaminants in heavily contaminated settings may reduce recovery rates of *E. coli.* Slaughter slabs have less adequate service modules in comparison to slaughterhouses in Ghana. Privately-owned slaughterhouses often have better slaughter infrastructure and compliance to hygienic practices in an effort to maintain customer satisfaction. This phenomenon may account for the low levels of *E. coli* contamination (9.5%) observed for the Privately-owned slaughterhouse.

The highest rate of resistance in *E. coli* was observed for ampicillin (57%). This follows a commonly reported pattern of beta- lactam antibiotic resistance rates of more than 50% in foods of animal origin [20, 21]. While these observations were attributed to the common use of ampicillin and penicillin derivatives in food animals, the scale of resistance is largely dependent on the food animal type and route of administration [22]. Sub-therapeutic doses of beta-lactams administered through feed and water in poultry and the pig industry may yield higher rates of resistance than in cattle where parenteral administration is common [23]. The levels of resistance to tetracycline (45%) and SXT (17%) observed in this study have similarly been reported in *E. coli* from beef in Ghana (44 and 18%) [24]. Tetracyclines are commonly used for therapy in humans and livestock and for growth promotion in intensive farming systems through feed [25]. High transferability of tetracycline resistance determinants in gut bacteria of livestock in such settings may contribute to the observed rates of tetracycline resistance in *E. coli* isolated from raw meat, meat products and the environment [26].

We documented lower rates of resistance of *E. coli* to cefuroxime in comparison to isolates recovered from clinical specimens in Ghana [27]. In comparison to its use in humans, second generation cephalosporins are less commonly used in food animals and are broadly approved for the treatment of mastitis in dairy cattle [28]. Among SXT resistant *E. coli* isolates of animal origin, ampicillin and tetracycline have been identified in previous studies as common co-transferred resistant phenotypes [29]. The MDR rate of 22% in *E. coli* isolates was similar to studies carried out by Saud et al. on raw buffalo meat while significantly higher rates were observed for chicken in the same study [30]. These findings can be attributed to the routine use of antibiotics in poultry feed and less common use of oral antibiotics in large animals. The detection of the *TEM* gene in four *E. coli* isolates is comparable to studies by Sheikh et al. that found a lower incidence of the *TEM* gene in ground beef as compared to other meat types [31]. Several factors may account for the low proportion of ESBL producing *E. coli* recovered in this study. Studies conducted by Aldeyab et al. posited that community incidence of ESBL-producing bacteria was significantly linked to amoxicillin-clavulanic acid use while hospital incidence was linked to fluoroquinolone use in health care settings [32]. ESBL production in *Enterobacteriaceae* recovered from foods of animal origin may reflect the burden of resistance to certain classes of antimicrobial drugs in gut bacteria in different food animals. The presence of ESBL-producing *E. coli* in raw meat may cause infections in humans and lead to prolonged hospital stays, increased medical costs, increased mortality and morbidity [33].

Our isolates showed high susceptibility rates to meropenem, ceftriaxone, chloramphenicol, and gentamycin. Third generation cephalosporins and phenicols are not commonly used in livestock in Ghana, thus their efficacy has been maintained [34]. Preserving the efficacy of these drugs is a necessary measure as they fall in the category of critically needed antibiotics used to treat life-threatening infections caused by *E. coli* in humans.

While this study provides evidence of the presence of drug resistant bacteria in raw meat, its interpretation is limited to known determinants of resistance in the primary production chain as environmental determinants such as water and soil were not assessed. Water used in the slaughter process may serve as a reservoir of antibiotic resistant bacteria and a constant source of contamination for raw meat. The study did not assess the contribution of soil microbiome at the study sites to contamination of raw meat with antibiotic resistant bacteria. Future studies that provide a holistic approach for determining the sources of raw meat contamination at slaughter sites are required to reduce the risk of spread of antibiotic resistant bacteria to humans through the food chain.

## Conclusions

Our study found that more than half of meat samples had unacceptable levels of microbial contamination. Contamination with *E. coli* was found in 48% of meat samples with multidrug resistance observed in 22% of isolates. Meat handlers at the selected slaughter sites and consumers are at risk of foodborne infections from *E. coli* including ESBL producing *E. coli* that are resistant at various levels to most antibiotics in use. This calls for further investigation of the sources of meat contamination and an assessment of hygiene standards in slaughterhouses in the Greater Accra Region. Control of potential hazards at slaughter is a necessary measure to minimize the incidence of contamination in meat. Enhanced surveillance in food products of animal origin is required to monitor trends in resistance patterns and promptly detect emerging resistant bacteria.

## Methods

### Study sites and sample collection

A cross-sectional study involving the collection of surface swab samples from meat was conducted at three slaughter sites in the Greater Accra region of Ghana from January 2019 to June 2019. The sites consisted of a public slaughterhouse, a slaughter slab and a privately-owned slaughterhouse. Cattle and goats were slaughtered daily at all three sites whereas sheep were slaughtered occasionally. Butchers operating at these slaughter sites obtained their livestock mainly from the three northern regions of Ghana. All three sites were points of meat sale and had the basic modules of production, which included a slaughter floor and lairage/holding pen. All parts of carcasses were sold as meat in large cuts and small cuts at these sites. On predetermined weekly sampling days, a random number was generated for the first sample after which systematic random sampling was used for the selection of up to 5 carcasses for each meat type. A total of 205 surface swab samples were collected from beef (*n* = 81), chevon (*n* = 108) and mutton (*n* = 16). Sample collection was done immediately after carcass dressing with a sterile cotton swab and a sterile template of size 100 cm^2^ for cattle and 25 cm^2^ for goat and sheep carcasses. For each beef carcass, surface swabs were collected from the thigh, brisket and flank and pooled into a single tube containing 10 ml of Buffered Peptone Water (BPW). Swabs from goat and sheep carcasses were obtained from thigh, brisket and mid-loin [35]. All samples were transported to the Bacteriology Department, Noguchi Memorial Institute for Medical Research, within 2 h of sample collection for processing.

### Enumeration of bacteria

Total plate counts and total coliform counts were performed for all samples using the pour plate method. Serial dilutions of ten-fold units of each sample were plated on Plate Count Agar (Oxoid) and incubated for 48 h at 30 °C. Following incubation, plates with colonies ranging from 30 to 300 were counted and expressed in CFU/cm^2^. The total coliform count was performed in the same way using Brilliance *E. coli*/ Coliform selective agar (Oxoid). The results of both counts were interpreted using guidelines set by the Ghana Standards Authority and the European Commission Regulation on the microbial criteria for food stuffs [36, 37]. Limits to total coliform counts were not specified in these documents hence limits set for *Enterobacteriaceae* counts in both documents were adopted for total coliform counts.

### Identification of *E. coli* in meat

To isolate *E. coli*, samples were pre-enriched in Brain Heart Infusion (BHI) at 37 °C for a period of 24 h. Ten (10 μl) microliters of each sample were then plated on MacConkey agar and incubated overnight at 37 °C. We identified *E. coli* by colony morphology and confirmed the isolates using Matrix Assisted Laser Desorption/Ionization -Time of Flight Mass Spectrometry (MALDI-TOF MS). Colonies from fresh overnight cultures were spotted on the MALDI-TOF MS target plate. One [1] μl of formic acid was then added and allowed to dry for 15 min. One [1] μl of matrix preparation was placed on each sample and left to dry for a further 15 min. MALDI-TOF MS was then conducted and ionization peaks (spectra) generated were matched against the integrated reference library of the MALDI system for confirmation of species of bacteria.

### Antimicrobial susceptibility testing

Antimicrobial susceptibility testing was done by the Kirby Bauer disk diffusion method using the following antimicrobial agents (Oxoid Ltd., Hampshire, UK): ampicillin (10μg), tetracycline (30μg), cefuroxime (30μg), cefotaxime (5μg), ceftriaxone (30μg), amikacin (30μg), gentamycin (10μg), chloramphenicol (30μg), ciprofloxacin (5μg), sulphamethoxazole-trimethoprim (25μg) and meropenem (10μg). The procedure consisted of the preparation of a standard inoculum (0.5 McFarland) and inoculation on Mueller Hinton (MH) agar plates for 18 h at 35 °C [38]. Interpretation of zone sizes was done using the EUCAST guideline [39]. *E. coli* ATCC 25922 was used to monitor the performance of the test. In this study, we defined multidrug resistance (MDR) as resistance to three or more classes of antimicrobial agents.

### Phenotypic and genotypic detection of ESBL production

The combination disk method was used to identify ESBL producing *E. coli*. Briefly, ceftazidime disks and ceftazidime combined with clavulanic acid disks were positioned 30 mm apart on the inoculated plates, and incubated overnight at a temperature of 37 °C. Isolates were classified as ESBL positive if the difference between the inhibition zone diameter of ceftazidime combined with clavulanic acid and diameter of the inhibition zone for the ceftazidime-only disk was ≥5 mm [40]. Cefotaxime disks and cefotaxime combined with clavulanic acid disks were used concurrently with ceftazidime for confirmation of ESBL production. DNA was extracted from presumptive colonies for the genotypic detection of ESBL production.

Bacterial DNA extraction consisted of placing pure colonies of overnight growth in 1 ml of distilled water. The mixture was boiled for 10 min and centrifuged for 5 min, at 1000 rotations per minute. Using the supernatant as a DNA template, PCR was done to detect ESBL resistance genes (*bla*_*TEM,*_ *bla*_*SHV,*_ and *bla*_*CTX-M*_). The final volume of 25 μl contained 2 μl DNA template, 10 mM of each primer (Table 4) [41], PCR grade water (10ul) and multiplex PCR master mix (13ul) (QIAGEN). Multiplex PCR was carried out in a thermal cycler with the following cycling conditions: 95 °C for 5 min, 35 cycles of 95 °C for 30 s, 60 °C for 30 s, 72 °C for 2 min, and a final extension lap at 72 °C for 10 min.

**Table 4:** Oligonucleotide primers used to detect ESBL genes in *E. coli* isolates

Genes	Primer sequences	Expected amplicon size (bp)
TEM-1	Forward: 5′-GAGACAATAACCCTGGTAAAT-3′ Reverse: 5′-AGAAGTAAGTTGGCAGCAGTG-3′	459
CTX-M	Forward: 5′-GAAGGTCATCAAGAAGGTGCG-3′ Reverse: 5′-GCATTGCCACGCTTTTCATAG-3′	560
SHV	Forward: 5′-GTCAGCGAAAAACACCTTGCC’ Reverse: 5′-GTCTTATCGGCGATAAACCAG-3’	383

### Statistical analysis

Descriptive statistics were performed on carriage rates of *E. coli* and their resistance patterns. The Chi-square test was used to assess differences in the proportion of *E .coli* detected in raw meat between the three slaughter sites. A Kruskal-Wallis test was performed to determine if there were significant differences in microbial counts in raw meat among the slaughter sites. Statistical analyses were performed at 95% confidence level using STATA version 15.0.

## Data Availability

The data sets used during the current study are available from the corresponding author on reasonable request.

## Abbreviations

ATCC: American type culture collection
CFU: Colony forming unit
EUCAST: European Committee on Antimicrobial Susceptibility Testing
ESBL: Extended spectrum beta lactamase
SXT: Sulphamethoxazole/trimethoprim
MDR: Multi-drug resistance
